# Comparing surgical and endoscopic resection approaches for colorectal neuroendocrine tumors within the diameter range of 10-20mm: an inverse probability weighting analysis based on the SEER database

**DOI:** 10.3389/fendo.2024.1378968

**Published:** 2024-03-27

**Authors:** Jinyi Xu, Ruikai Liang, Qi Cai, Yang Liu, Xinyi Ge, Bin Lai, Shengxun Mao, Jiaqing Cao, Jiwei Wang

**Affiliations:** ^1^ Department of Gastrointestinal Surgery, The Second Affiliated Hospital, Jiangxi Medical College, Nanchang University, Nanchang, Jiangxi, China; ^2^ The Second Clinical Medical College of Nanchang University, The Second Affiliated Hospital of Nanchang University, Nanchang, Jiangxi, China; ^3^ Department of Ultrasound, The Second Affiliated Hospital, Jiangxi Medical College, Nanchang University, Nanchang, Jiangxi, China

**Keywords:** colorectal neuroendocrine tumor, endoscopic resection, surgical resection, SEER database, inverse probability weighting

## Abstract

**Background:**

Currently, the primary treatment modalities for colorectal neuroendocrine tumors (CRNET) with a diameter between 10mm and 20mm are surgical resection (SR) and endoscopic resection (ER). However, it remains unclear which surgical approach yields the greatest survival benefit for patients.

**Methods:**

This study included data from patients diagnosed with CRNET with tumor diameters ranging from 10mm to 20mm between the years 2004 and 2019, obtained from the Surveillance, Epidemiology, and End Results (SEER) database. Patients were categorized into ER and SR groups based on the respective surgical approaches. Inverse probability weighting (IPTW) was employed to mitigate selection bias. Kaplan-Meier analysis and log-rank tests were utilized to estimate overall survival (OS) and cancer-specific survival (CSS). Cox regression analysis (univariate and multivariate) was performed to evaluate potential factors influencing survival.

**Results:**

A total of 292 CRNET patients were included in this study (ER group: 108 individuals, SR group: 184 individuals). Prior to IPTW adjustment, Kaplan-Meier analysis and Cox proportional hazard regression analysis demonstrated that the OS and CSS of the SR group were inferior to those of the ER group. However, after IPTW adjustment, no statistically significant differences in prognosis were observed between the two groups. Subgroup analyses revealed that patients with muscular invasion, positive lymph nodes, or distant metastasis derived greater survival benefits from SR. Significant differences in OS and CSS between the two groups were also observed across different age groups.

**Conclusion:**

For patients with mucosal-limited lesions and without local lymph node or distant metastasis, ER is the preferred surgical approach. However, for patients with muscular invasion or positive lymph nodes/distant metastasis, SR offers a better prognosis. The choice of surgical approach should be based on the specific clinical characteristics of patients within different subgroups.

## 1 Introduction

Neuroendocrine tumors (NETs) originate from neuroendocrine cells and are a distinct type of heterogeneous tumor characterized by the production of biologically active amines and/or peptide hormone (1). The gastrointestinal tract is one of the most common sites for NETs occurrence, with colorectal NETs (CRNETs) being frequently reported. The incidence rate of CRNETs accounts for over one-third of the overall incidence rate of gastrointestinal NETs (2, 3). In recent years, the incidence rate of CRNETs has shown a significant upward trend worldwide. In the United States, the diagnostic rate of CRNETs is 1.4/100,000 (4, 5). The incidence rate of rectal NETs in Asian populations has risen from 0.2/100,000 in 1973 to 0.86/100,000 in 2004, which is significantly higher than that of Caucasians (6). Furthermore, the incidence rate of CRNETs is growing at the fastest rate among all NETs, accounting for 32.6% of all NETs and becoming the second most common NET in China (7). A study conducted in the Netherlands showed that the incidence rate of CRNETs increased from 0.36 cases per 100,000 residents in 2006 to 0.75 cases per 100,000 residents in 2011 (8). A national survey in Japan indicated that the prevalence of hindgut NETs (including CRNETs) rose from 2.07/100,000 in 2005 to 4.52/100,000 in 2010 (9).

Currently, the treatment options for CRNETs primarily involve endoscopic resection (ER) or surgical resection (SR) based on staging, while unresectable cases require medical therapy (10). For CRNETs with a diameter smaller than 10mm, ER treatment is typically recommended (11), whereas for larger lesions, partial or total intestinal resection through SR is chosen to reduce recurrence rates and improve patient prognosis (12, 13). However, the optimal treatment strategy for CRNETs with a diameter between 10-20mm has not been definitively determined. Previous studies have suggested that for lesions restricted to the submucosal layer, regardless of their diameter, ER can effectively achieve curative resection with minimal recurrence rates during follow-up (14). On the other hand, there are reports indicating a correlation between tumors larger than 15mm and a higher risk of distant metastasis (15). The NCCN guidelines also emphasize the need for examination under anesthesia or endoscopic ultrasound evaluation before surgery for tumors measuring 10-20mm (16).

Due to the rarity of CRNETs within the 10-20mm diameter range and the lack of relevant prospective studies, conflicting viewpoints have emerged based on studies with small sample populations. The impact of different surgical approaches on the prognosis of these tumors remains unclear. Therefore, our study aims to evaluate the long-term prognosis differences between CRNETs patients who undergo ER and SR. To accomplish this objective, we have included multicenter population data from the Surveillance, Epidemiology, and End Results (SEER) database. Inverse probability weighting (IPTW) is utilized to reduce selection bias and minimize interference from confounding factors.

## 2 Materials and methods

### 2.1 Data source

The patient cohort for this study was extracted from the SEER cancer database of the National Cancer Institute using SEER*Stat software (version 8.4.2). SEER is a population-based collection of multiple cancer registries covering approximately 28% of the U.S. population. As patient data in the SEER database are de-identified, ethical approval was not required for this study.

### 2.2 Patient selection criteria

This study included patients diagnosed with CRNET between the years 2004 and 2019. The inclusion criteria were as follows: [1] primary tumor site in the colon or rectum, [2] histologic codes according to the third edition of International Classification of Diseases for Oncology (ICD-O-3): 8012-8013, 8041-8044, 8240-8246, and 8249, [3] history of primary tumor resection, and [4] tumor diameter ranging from 10-20mm. Patients meeting any of the following criteria were excluded from the study: [1] survival time of 0, [2] missing clinical demographic information, and [3] cases provided through autopsy or death certificates.

### 2.3 Cohort definition and variable recode

Based on the different surgical approaches, all eligible CRNET patients were divided into ER and SR cohorts, then IPTW was performed to obtain more comparable cohorts (17). The analyzed variables included the following factors: age (≤70 or >70), gender (female or male), race (Black, White, or other), grade of differentiation (Grade I: well-differentiated; Grade II: moderately differentiated; Grade III: poorly differentiated; Grade IV: undifferentiated), T stage, N stage, M stage, tumor location (colon or rectum), radiotherapy (yes or no), and chemotherapy (yes or no). The main outcomes of the study were overall survival (OS) and cancer-specific survival (CSS). The optimal cutoff values for continuous variables were determined using x-tile software analysis. In this study, TNM staging was based on the data at the time of diagnosis and followed the 8th edition of the AJCC staging system for colorectal cancer.

### 2.4 Statistical analyses

This study is a retrospective observational study; therefore, the allocation of surgical approaches was non-random. IPTW was used to adjust for clinical baseline covariate differences between the two intervention groups and eliminate selection bias (18). Chi-square tests or Student’s t-tests were used to assess demographic characteristics before and after matching, with a P-value >0.05 indicating no statistically significant differences between the two groups. OS and CSS of the population in both cohorts were compared using log-rank tests (survdiff function for pre-IPTW data and svylogrank function for weighted data) and illustrated using Kaplan-Meier curves. In the matched population, univariate and multivariate Cox proportional hazards regression models were employed to identify independent prognostic factors associated with OS in CRNET patients. Additionally, subgroup analyses using univariable Cox regression were conducted within each variable subgroup to investigate the impact of the two surgical approaches on OS and CSS. All statistical analyses and visualizations in this study were performed using R software (version 4.3.1). Two-sided P-values <0.05 were considered statistically significant.

## 3 Results

### 3.1 Baseline characteristics of the study population

After selection, a total of 627 eligible CRNET patients were included in this study, with 319 in the ER group and 308 in the SR group. As shown in **Table 1**, significant differences in baseline characteristics existed between the two groups in the unweighted data. Compared to the SR group, the ER group had a younger mean age (56.14 ± 11.38 vs 63.40 ± 12.32), lower proportions of patients receiving chemotherapy (6.0% vs 20.5%) and radiotherapy (2.5% vs 7.5%), as well as a higher proportion of patients in earlier TNM stages and Grade levels. After IPTW adjustment, all variables had P-values greater than 0.05, indicating that the baseline characteristics between the two groups were essentially similar with no statistical differences (**Table 1**).

**Table 1:** Clinical baseline characteristics of patients in the endoscopic resection and surgical resection groups before and after IPTW adjustment.

Characteristic	Unmatched			IPTW		
	Endoscopic Resection	Surgical Resection	P	Endoscopic Resection	Surgical Resection	P
	(n=319)	(n=308)				
**Age (mean (SD))**	56.14 (11.38)	63.40 (12.32)	<0.001	57.76 (11.34)	60.02 (12.48)	0.126
**Tumor_size (mean (SD))**	12.81 (3.21)	15.68 (3.47)	<0.001	13.70 (3.48)	14.59 (3.60)	0.115
**Sex (%)**			0.999			0.727
Female	174 (54.5)	169 (54.9)		287.3 (54.4)	355.7 (56.8)	
Male	145 (45.5)	139 (45.1)		240.8 (45.6)	270.7 (43.2)	
**Race (%)**			<0.001			0.925
White	163 (51.1)	235 (76.3)		337.2 (63.8)	414.5 (66.2)	
Black	88 (27.6)	47 (15.3)		113.4 (21.5)	124.4 (19.9)	
Other	68 (21.3)	26 (8.4)		77.6 (14.7)	87.4 (14.0)	
**Grade (%)**			<0.001			0.598
Grade I	189 (59.2)	149 (48.4)		269.3 (51.0)	342.0 (54.6)	
Grade II	35 (11.0)	47 (15.3)		51.5 (9.8)	94.4 (15.1)	
Grade III	16 (5.0)	53 (17.2)		89.5 (16.9)	69.8 (11.1)	
Grade IV	7 (2.2)	27 (8.8)		33.6 (6.4)	34.3 (5.5)	
Unknown	72 (22.6)	32 (10.4)		84.3 (16.0)	85.9 (13.7)	
**T_stage (%)**			<0.001			0.424
Tis	15 (4.7)	1 (0.3)		16.0 (3.0)	11.5 (1.8)	
T1	273 (85.6)	86 (27.9)		365.8 (69.3)	362.4 (57.9)	
T2	23 (7.2)	69 (22.4)		67.9 (12.9)	92.6 (14.8)	
T3	7 (2.2)	114 (37.0)		70.5 (13.3)	120.9 (19.3)	
T4	1 (0.3)	38 (12.3)		8.0 (1.5)	38.9 (6.2)	
**N_stage (%)**			<0.001			0.094
N0	309 (96.9)	116 (37.7)		423.2 (80.1)	424.7 (67.8)	
N1	7 (2.2)	142 (46.1)		98.1 (18.6)	148.9 (23.8)	
N2	3 (0.9)	50 (16.2)		6.9 (1.3)	52.7 (8.4)	
**M_stage (%)**			<0.001			0.352
M0	303 (95.0)	259 (84.1)		425.7 (80.6)	545.5 (87.1)	
M1	16 (5.0)	49 (15.9)		102.5 (19.4)	80.8 (12.9)	
**Radiotherapy (%)**			0.007			0.522
No	311 (97.5)	285 (92.5)		475.8 (90.1)	587.2 (93.8)	
Yes	8 (2.5)	23 (7.5)		52.3 (9.9)	39.1 (6.2)	
**Chemotherapy (%)**			<0.001			0.449
No	300 (94.0)	245 (79.5)		417.0 (78.9)	528.8 (84.4)	
Yes	19 (6.0)	63 (20.5)		111.2 (21.1)	97.6 (15.6)	

### 3.2 Survival analyses

**Figure 1:**
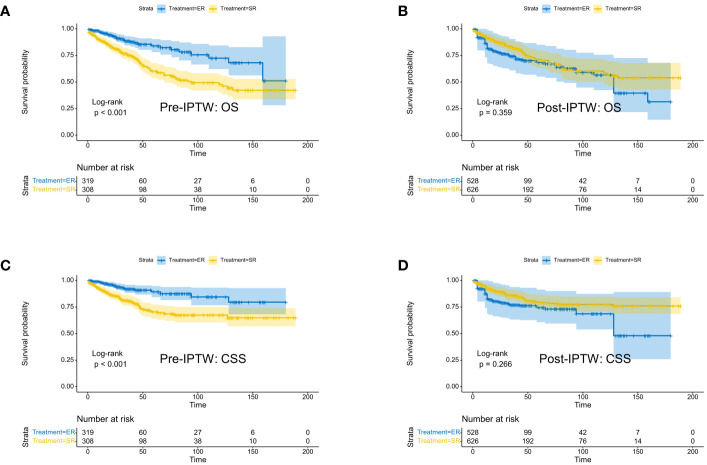
Kaplan-Meier survival curves of CRNET patients undergoing surgical resection and endoscopic resection in the study population before IPTW (**A**: OS; **C**: CSS) and after IPTW (**B**: OS; **D**: CSS).

Kaplan-Meier analysis (**Figure 1**) demonstrated that in the original population, ER group patients had significantly better overall survival (OS) (p<0.001) and cancer-specific survival (CSS) (p<0.001) than SR group patients. However, after IPTW adjustment, there were no statistically significant differences in prognosis between the two groups (OS: p=0.359; CSS: p=0.266). In the unweighted cohort, the multivariable Cox proportional hazards regression model revealed that independent prognostic factors for OS in CRNET patients were Age, Grade, N stage, M stage, and radiotherapy. The IPTW-adjusted multivariable Cox proportional hazards regression model showed that independent prognostic factors for OS in CRNET patients remained Age, Grade, N stage, M stage, and radiotherapy (**Table 2**). Surgical approach and tumor location did not show statistical significance (p>0.05).

**Table 2:** Univariate and multivariate Cox regression analyses of overall survival in CRNET patients before and after IPTW adjustment.

Characteristic	Unadjusted				IPTW-adjusted			
	Univariate analysis		Multivariate analysis		Univariate analysis		Multivariate analysis	
	HR (95% CI)	P	HR (95% CI)	P	HR (95% CI)	P	HR (95% CI)	P
Age								
≤70	Reference		Reference		Reference		Reference	
>70	3.969(2.839-5.549)	<0.001^*^	3.179(2.191-4.613)	<0.001^*^	2.692(1.521-4.766)	<0.001^*^	2.850(1.781-4.557)	<0.001^*^
Sex								
Female	Reference				Reference			
Male	1.241(0.888-1.735)	0.206			1.597(0.779-3.273)	0.201		
Race								
White	Reference				Reference			
Black	0.697(0.443-1.097)	0.119			0.447(0.229-0.875)	0.019^*^	1.516(0.787-2.920)	0.213
Other	0.645(0.362-1.150)	0.137			0.349(0.153-0.794)	0.012^*^	0.873(0.445-1.712)	0.692
Grade								
I	Reference		Reference		Reference		Reference	
II	2.008(1.155-3.493)	0.014^*^	1.471(0.836-2.588)	0.181	1.470(0.685-3.156)	0.323	1.063(0.465-2.429)	0.885
III	4.971(3.181-7.767)	<0.001^*^	2.701(1.617-4.511)	<0.001^*^	7.137(3.201-15.913)	<0.001^*^	3.263(1.781-5.977)	<0.001^*^
IV	7.393(4.390-12.451)	<0.001^*^	7.258(4.054-12.993)	<0.001^*^	8.287(3.743-18.345)	<0.001^*^	7.261(3.556-14.826)	<0.001^*^
Unknown	1.695(0.954-3.012)	0.072	1.942(1.061-3.552)	0.031^*^	1.395(0.604-3.222)	0.436	1.567(0.714-3.437)	0.263
T stage								
Tis	Reference				Reference		Reference	
T1	1.725(0.536-5.554)	0.361			3.406(0.734-15.810)	0.118	1.330(0.238-7.426)	0.745
T2	2.265(0.674-7.641)	0.186			5.413(1.059-27.680)	0.043^*^	1.530(0.271-8.630)	0.630
T3	3.205(0.992-10.354)	0.052			8.799(1.783-43.420)	0.008^*^	1.770(0.316-9.911)	0.516
T4	5.015(1.487-16.910)	0.009^*^			6.799(1.343-34.430)	0.021^*^	1.327(0.222-7.923)	0.757
N stage								
N0	Reference		Reference		Reference		Reference	
N1	2.030(1.366-3.017)	<0.001^*^	1.947(1.228-3.087)	0.005^*^	4.866(2.486-9.522)	<0.001^*^	2.558(1.601-4.086)	<0.001^*^
N2	3.847(2.535-5.837)	<0.001^*^	2.766(1.661-4.606)	<0.001^*^	4.492(2.789-7.236)	<0.001^*^	1.994(1.080-3.679)	0.027^*^
M stage								
M0	Reference		Reference		Reference		Reference	
M1	4.959(3.443-7.142)	<0.001^*^	4.825(3.179-7.322)	<0.001^*^	9.136(5.032-16.590)	<0.001^*^	7.879(4.835-12.840)	<0.001^*^
Radiotherapy								
No	Reference		Reference		Reference		Reference	
Yes	3.075(1.849-5.113)	<0.001^*^	2.033(1.141-3.623)	0.016^*^	5.224(1.762-15.480)	0.003^*^	3.494(1.733-7.047)	<0.001^*^
Chemotherapy								
No	Reference		Reference		Reference		Reference	
Yes	3.156(2.222-4.484)	<0.001^*^	0.727(0.470-1.124)	0.152	5.059(2.615-9.786)	<0.001^*^	0.766(0.478-1.226)	0.267
Site								
Colon	Reference				Reference			
Rectum	0.602(0.426-0.852)	0.004^*^	1.387(0.809-2.379)	0.234	1.174(0.665-2.073)	0.579		
Type of surgery								
ER	Reference		Reference		Reference			
SR	2.473(1.688-3.623)	<0.001^*^	1.117(0.617-2.024)	0.715	0.706(0.371-1.343)	0.289		

*A P-value less than 0.05 has statistical significance.

### 3.3 Subgroup analysis

**Figure 2:**
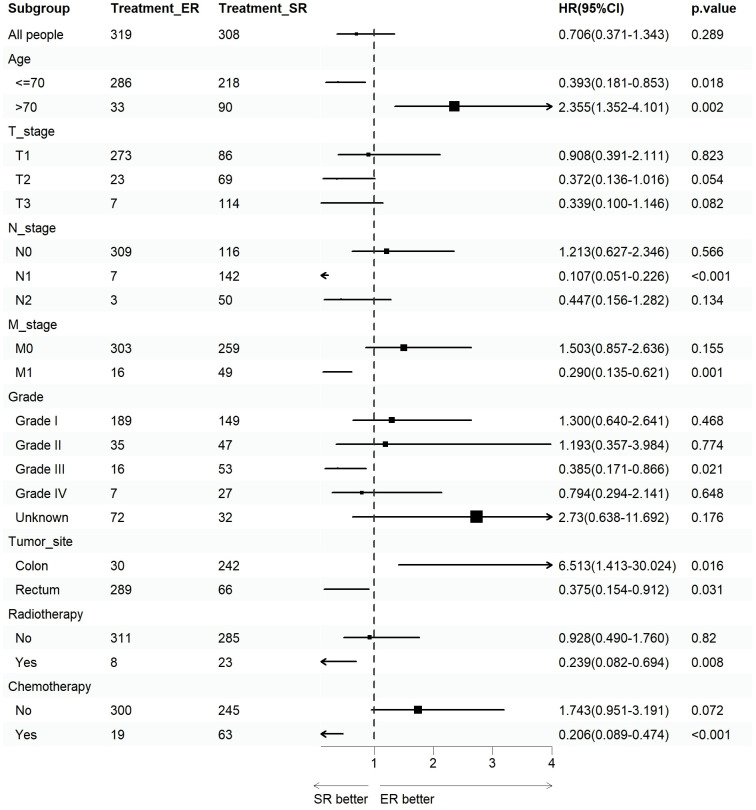
Forest plot of patients with CRNET included in the OS subgroup analysis (endoscopic resection vs. surgical resection).

**Figure 3:**
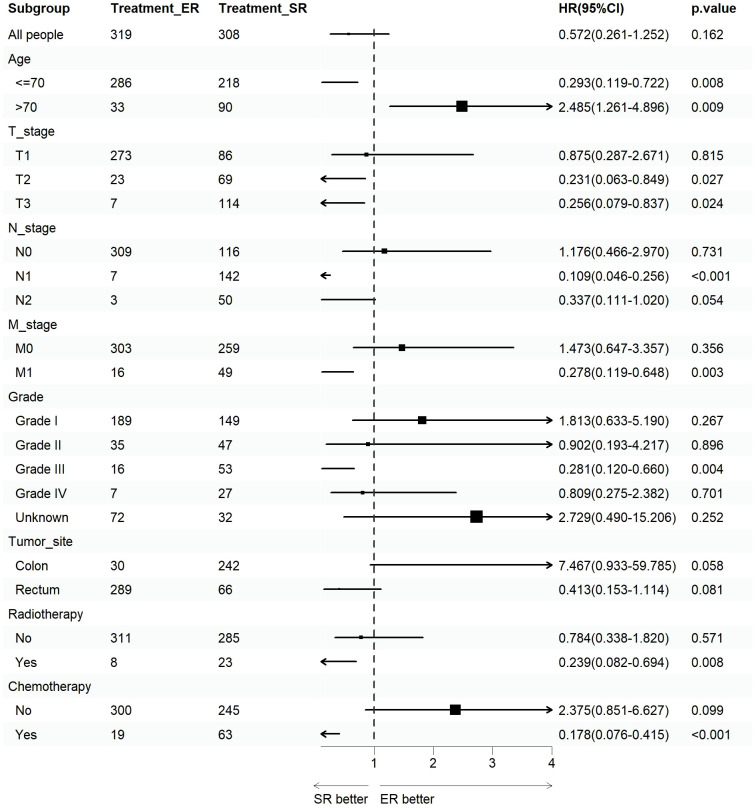
Forest plot of patients with CRNET included in the CSS subgroup analysis (endoscopic resection vs. surgical resection).

**Figure 4:**
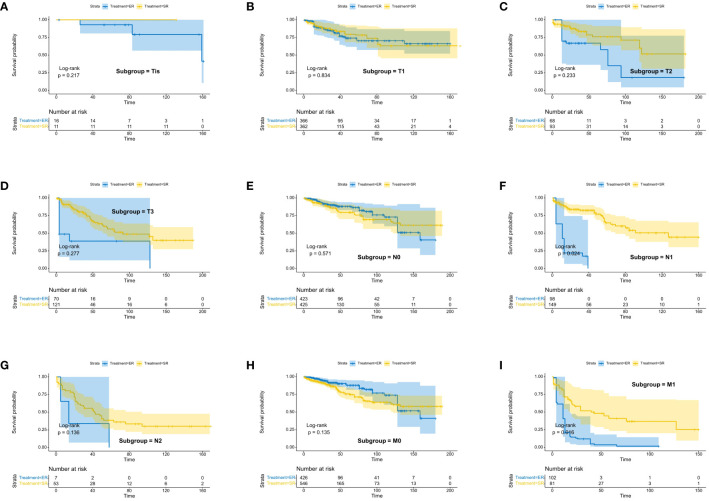
Kaplan-Meier estimates of OS with corresponding 95% confidence intervals for patients in the endoscopic resection and surgical resection groups after IPTW: Stratified by T stage **(A–D)**, N stage **(E–G)**, M-Stage **(H, I)**.

**Figure 5:**
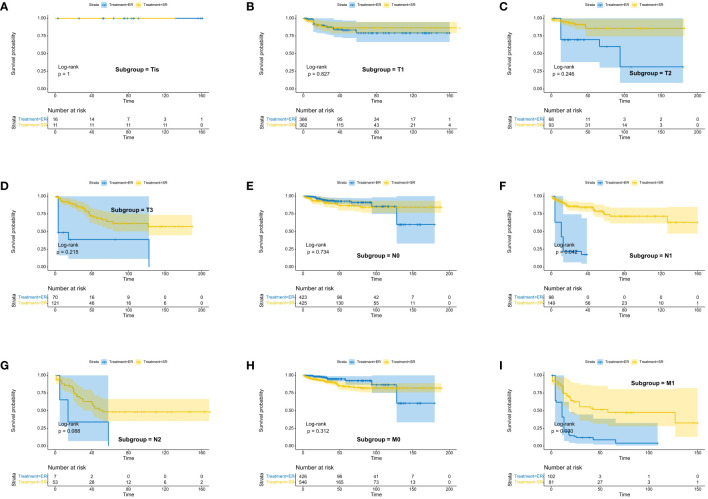
Kaplan-Meier estimates of CSS with corresponding 95% confidence intervals for patients in the endoscopic resection and surgical resection groups after IPTW: Stratified by T stage **(A–D)**, N stage **(E–G)**, M-Stage **(H, I)**.

We assessed the benefits of different surgical approaches in various subgroups and presented the prognostic differences between the SR and ER groups using a forest plot (**Figures 2**, **3**). The results showed that in the subgroup aged 70 years or younger, SR group had a better OS and CSS compared to ER group (OS: HR=0.393, p=0.018; CSS: HR=0.293, p=0.008). Conversely, in the subgroup aged over 70 years, the opposite trend was observed (OS: HR=2.355, p=0.002; CSS: HR=2.485, p=0.009). Grade did not influence the benefits of different surgical approaches. We found no statistically significant differences in OS and CSS between the SR and ER groups in the subgroups of T1, N0, and M0 (P>0.05). However, in the subgroups of T2 and T3, the CSS was better in the SR group compared to the ER group (T2: HR=0.231, P=0.027; T3: HR=0.256, P=0.024), while there was no statistically significant difference in OS between the two groups (P>0.05) (Due to the small number of patients in the Tis and T4 subgroups and the presence of extreme cases, which led to model overfitting, they were not displayed in the forest plot). Similarly, in the N1 subgroup, both OS and CSS were superior in the SR group compared to the ER group (OS: HR=0.107, P<0.001; CSS: HR=0.109, P<0.001), but there was no statistically significant difference in OS and CSS between the SR and ER groups in the N2 subgroup (P>0.05). We believe that the reason for these findings is the small number of cases in the T2, T3, and N2 subgroups, which resulted in statistical bias. Therefore, we further plotted the KM survival curves for each subgroup in the TNM staging to demonstrate the survival trends of patients in the SR and ER groups (**Figures 4**, **5**). Despite the statistical bias caused by the small number of cases, we still observed that patients in the SR group had better survival trends than those in the ER group in the T2, T3, and N2 subgroups. Thus, we have reason to believe that SR can bring better survival benefits in the T2-T4, N1-N2, and M1 subgroups.

## 4 Discussion

Rectal NETs account for approximately 1-2% of all rectal tumors, while the proportion of colon NETs is even lower. Moreover, cases of CRNET with a tumor diameter between 10-20mm are even rarer (19). Therefore, there is currently a lack of accurate research regarding the choice between ER and SR treatment for CRNET in the 10-20mm range. Although several studies suggest that ER may be a preferable choice for these patients (20, 21), there are still discrepancies in the specific treatment details among different subgroups. Additionally, a retrospective study has indicated that approximately 66% of these patients present with metastasis (14), which should remind us to choose treatment approaches more cautiously.

In this study, we analyzed representative multicenter data from the SEER database to assess the differences in benefits between ER and SR in CRNET patients with tumor diameters ranging from 10-20mm. Considering that intestinal resection can significantly impact patients’ quality of life and have irreversible effects on normal physiological activities, we selected OS as the primary outcome measure and also explored the differences in CSS. Before IPTW adjustment, the OS and CSS of patients undergoing SR were inferior to those undergoing ER, mostly due to selection bias related to higher tumor stages in the SR group. However, after IPTW adjustment, there were no statistically significant differences in OS and CSS between the two groups. In subgroup analyses, we found that patients aged 70 or younger benefited more from SR, while those over 70 derived more benefits from ER. We hypothesize that this could be attributed to the higher rates of lymph node and distant metastasis in younger patients (22–24).

We observed no statistically significant differences in OS and CSS between the two surgical approaches for patients with lesions limited to the mucosal layer. However, patients with muscular invasion were better suited for SR. We also noted that there were no differences in OS and CSS between the two procedures for patients with T3 and T4 stages. However, based on the trend revealed by KM survival curves, we believe this may be due to statistical biases resulting from a small sample size. Therefore, we suggest that patients with lesions invading beyond the mucosal layer should consider SR to reduce postoperative recurrence rates and improve prognosis. Previous studies have raised similar points: for 10-19mm rectal NETs without muscular or lymphatic vessel invasion, there is no association between curative surgical resection and higher radical resection rates (25). However, in the NCCN guidelines released in August 2023, it is recommended that for Grade I/II rectal NET patients with tumor diameters smaller than 20mm and without lymph node or distant metastasis, invasive depth should not be considered, and ER can be an option. Consistent with most previous studies (26), our findings from subgroup analyses and KM survival curves lead us to believe that patients with lymph node and distant metastasis benefit more from SR. Additionally, we do not consider Grade grading as an independent factor for surgical approach selection in CRNET patients with a diameter of 10-20mm.

In our study cohort, we found that current clinical practice tends to favor SR for patients with lesions located in the colon. Since the incidence of rectal NETs is significantly higher than that of colon NETs, research has mainly focused on rectal NET patients, while standardized surgical approaches for colon NETs remain lacking. In our study, we discovered that tumor location does not significantly impact the prognosis of CRNET patients, and considering long-term outcomes, patients with lesions in the colon benefit more from ER. For patients with colon NETs, the extent of intestinal resection is usually larger, which irreversibly affects patients’ quality of life. Thus, when R0 resection can be achieved through ER (27), recommending ER is a wiser option.

Furthermore, we identified age, N stage, M stage, and Grade grading as independent prognostic factors for the target patients. Therefore, we emphasize the importance of comprehensive preoperative evaluations for CRNET patients with tumor diameters between 10-20mm. As some studies have indicated, approximately 4%-20% of patients with tumors below 20mm in diameter may experience synchronous or metachronous metastasis (28), and there are significant changes in the risk of metastasis when the tumor diameter exceeds 15mm (15, 29, 30). A recent multicenter study demonstrated that a tumor diameter larger than 11.5mm is an independent risk factor for lymph node metastasis, and once lymph node metastasis occurs, ER may no longer be suitable (31). This study further lowered the clinical decision threshold for tumor diameter to 11.5mm, highlighting the need for cautious selection of diagnostic and treatment strategies for CRNET patients with tumor diameters of 10-20mm. Additionally, ENETS guidelines explicitly recommend baseline local staging through pelvic MRI for all rectal NETs larger than 10mm and chest CT, abdominal CT/MRI, and 68Ga-SSR-PET/CT evaluations for assessing distant metastasis (32).Considering these reasons, we strongly recommend that patients undergo thorough preoperative local and systemic examinations, including endoscopy under ultrasound guidance and relevant imaging evaluations, to select more accurate treatment approaches based on comprehensive assessments.

Currently, there are various subcategories of ER treatment for CRNET patients, including endoscopic mucosal resection (EMR), modified EMR (mEMR), endoscopic submucosal dissection (ESD), endoscopic submucosal resection with a ligation device (ESMR-L), and the more recent full-thickness endoscopic resection (EFTR) (33). The optimal endoscopic resection technique for small localized CRNETs has been a topic of controversy. In a recent retrospective study, Hamada et al. proposed that ESMR-L and ESD are the two best endoscopic techniques for treating rectal NETs with diameters less than 10mm and 10-14mm, respectively (34). Their findings also confirmed the safety and efficacy of endoscopic resection for small NETs (33). Moreover, EFTR has gained increasing popularity among clinicians, as it allows for complete removal of lesions, including surrounding layers of the intestinal wall, providing more thorough resection for NETs growing beneath the mucosal layer (33). However, there is currently a lack of clinical studies confirming its clinical benefits.

In this study, we incorporated multi-center data and conducted subgroup analysis to comprehensively explore the benefits of ER and SR in CRNET patients with tumor diameters of 10-20mm. Although we used IPTW to adjust for baseline characteristics and eliminate selection bias, providing relatively reliable conclusions, we must acknowledge certain limitations of this study. Firstly, the lack of important data such as Ki-67 index and mitotic count in the SEER database prevented precise grading of NETs. Additionally, due to the absence of specific information on radiotherapy, chemotherapy, and treatment with somatostatin analogs, the analysis of prognostic factors for NET patients using the SEER data is considerably limited (35). Secondly, due to the low incidence rate of NETs and our screening criteria based on tumor diameter, the number of patients included in the study remains relatively small, despite using multi-center population data. Thirdly, as this study involved retrospective analysis, biases may arise from the exclusion of cases with incomplete information.

## 5 Conclusion

There is no significant difference in prognosis between SR and ER in all CRNET patients with tumor diameters ranging from 10-20mm. For patients with mucosal-limited lesions and without local lymph node or distant metastasis, ER is the preferred surgical approach. However, for patients with muscular invasion or positive lymph nodes/distant metastasis, SR offers a better prognosis. The choice of surgical approach should be based on the specific clinical characteristics of patients within different subgroups.

## Data availability statement

Publicly available datasets were analyzed in this study. This data can be found here: https://seer.cancer.gov/.

## Ethics statement

Since the data from SEER are publicly available and deidentified, this study was exempt from local institutional review board review.

## Author contributions

JX: Writing – original draft, Software, Methodology, Investigation, Formal analysis, Data curation, Conceptualization. RL: Writing – original draft, Software, Methodology, Formal analysis. QC: Writing – original draft, Validation, Project administration, Formal analysis. YL: Writing – original draft, Software, Resources, Data curation. XG: Writing – original draft, Formal analysis, Data curation. BL: Writing – review & editing, Visualization, Validation, Supervision. SM: Writing – review & editing, Resources, Project administration. JC: Writing – review & editing, Supervision, Resources. JW: Writing – review & editing, Visualization, Validation, Resources, Funding acquisition.
